# A Case of Paragonimiasis in a Patient with Wet Cough

**DOI:** 10.4269/ajtmh.20-0395

**Published:** 2020-09

**Authors:** Kotaro Kunitomo, Shinya Yumoto, Takahiro Tsuji

**Affiliations:** 1 Department of Internal Medicine, Kumamoto Medical Center, Kumamoto, Japan;; 2 Department of Internal Medicine, Aso Medical Center, Kumamoto, Japan

**Figure 1.**
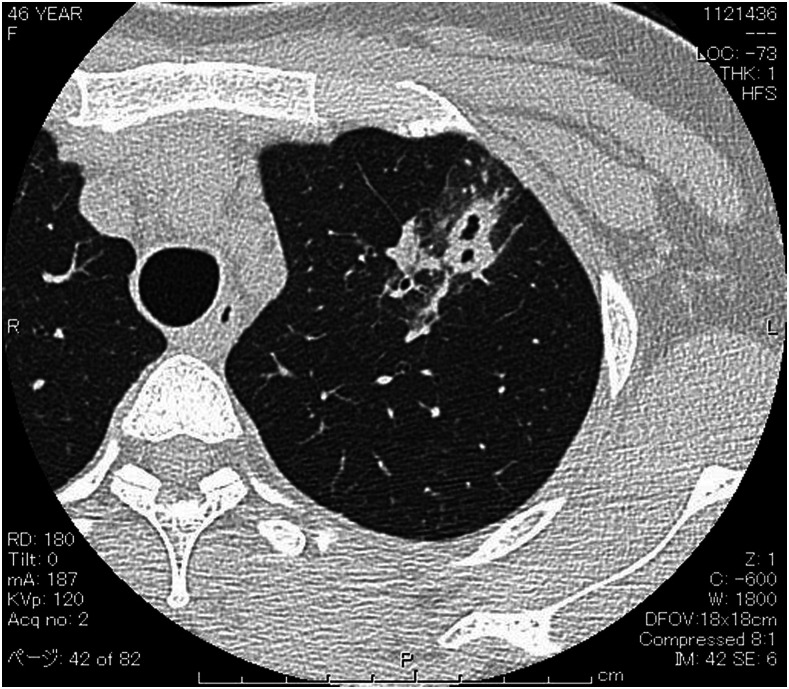
Chest computed tomography.

A 46-year-old Chinese woman living in Japan presented with wet cough that persisted for 20 days. She had traveled to Cambodia 50 days prior, where she consumed freshwater crabs. She had not ingested raw crab or crayfish in Japan. Her temperature was 36.9°C; blood pressure, 127/88 mmHg; respiratory rate, 14 breaths/minutes; pulse, 86 beats/minutes; and oxygen saturation, 98% at rest while breathing ambient air. Chest examination revealed no crackles; physical examination results were otherwise unremarkable. Laboratory investigations revealed a white blood cell count of 7.0 × 10^9^/L (eosinophils: 5%). Chest computed tomography showed a nodular shadow with a cavity and a surrounding ground-glass appearance in the left upper lung lobe (Figure 1).

**Figure 2.**
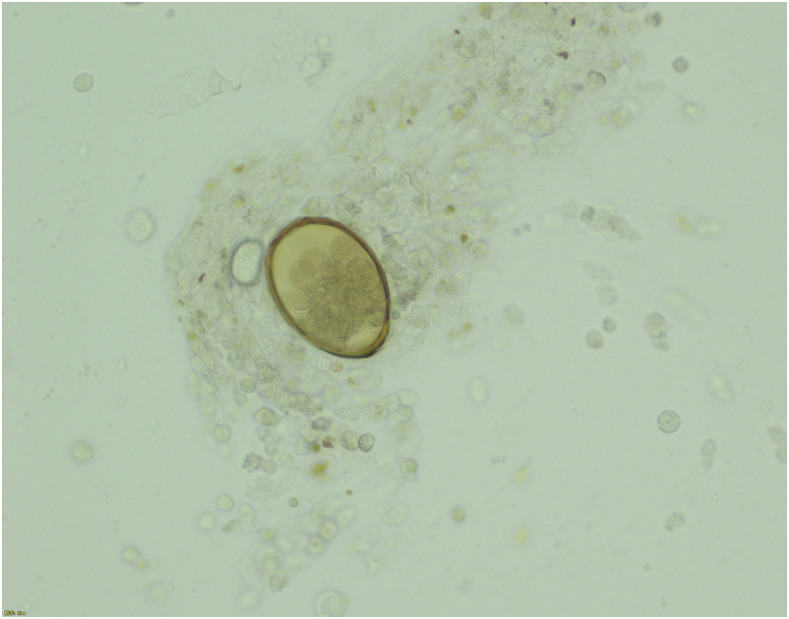
Bronchoalveolar lavage smears. This figure appears in color at www.ajtmh.org.

Bronchoalveolar lavage smears were negative for acid-fast bacilli and fungi, and no malignant cells were observed on cytological examination. However, microscopic examination of bronchoalveolar lavage fluid revealed brown oval eggs, suggesting the presence of *Paragonimus westermani* (Figure 2). The final diagnosis was paragonimiasis (*P. westermani*) because the serologic antibody test was positive for *P. westermani* (IgG), and she was treated with praziquantel. Her clinical symptoms improved within 2 months.

*Paragonimus westermani* is a parasite of crustaceans, prevalent in Asia, transmitted to humans via the consumption of raw or poorly cooked freshwater crabs. There are about 30–50 cases of lung infections diagnosed in Japan every year.^1^ The symptoms of paragonimiasis include chronic cough, chest pain, dyspnea, and hemoptysis, which are similar to those of tuberculosis and lung cancer.^2^ Paragonimiasis is diagnosed by confirming the presence of eggs in patients’ sputum or fecal samples.^3^ Typical computed tomography findings of paragonimiasis are focal pleural thickening and subpleural linear opacities, leading to the development of necrotic peripheral pulmonary nodules.^4^ Praziquantel is the treatment of choice.^2^ The frequency of parasitic diseases, including paragonimiasis, may increase worldwide, considering global dietary habit changes and increasing international travel.
